# Investigation of Nano Spray-Dried, Hyaluronic Acid-Modified Polymeric Micelles for Nasal Administration

**DOI:** 10.3390/pharmaceutics17040533

**Published:** 2025-04-18

**Authors:** Bence Sipos, Levente Mayer, Mária Budai-Szűcs, Gábor Katona, Rita Ambrus, Ildikó Csóka

**Affiliations:** Institute of Pharmaceutical Technology and Regulatory Affairs, University of Szeged, Eötvös Street 6, H-6720 Szeged, Hungary; mayer.levente56@gmail.com (L.M.); budai-szucs.maria@szte.hu (M.B.-S.); katona.gabor@szte.hu (G.K.); ambrus.rita@szte.hu (R.A.); csoka.ildiko@szte.hu (I.C.)

**Keywords:** polymeric micelle, nasal administration, nanomedicine, hyaluronic acid, nano spray-drying

## Abstract

**Background/Objectives**: The combination of nanomedicine with nasal administration is of paramount importance in current research and development. Polymeric micelles coated with hyaluronic acid may be a suitable solution to enhance drug release and permeation whilst properly adhering to the nasal mucosa, increasing residence time. **Methods**: Solid state characterization included morphology and laser diffraction-based size analysis and X-ray powder diffraction. The characterization of dispersed polymeric micelles in aqueous media was performed based on dynamic light scattering and determining the solubility enhancement related factors such as encapsulation efficiency and thermodynamic solubility. In vitro nasal drug release and permeability studies were also conducted to characterize the different hyaluronic acid-modified polymeric micelles. Quantitative measurements were carried out via liquid chromatography. **Results**: Concentration dependence on hyaluronic acid was found during all measurements, with one formulation candidate overcoming the others. With a high yield above 80%, monodispersed particles were formulated with an approximately 4 µm particle size in uniform distribution and spherical morphology. The small micelle size (107.3 nm) in uniform manner led to a high encapsulation efficiency above 80% and released the drug amount above 70% in 15 min. High drug permeation was also achieved compared with the initial active substance by itself. **Conclusions**: A value-added polymeric micelle formulation was developed with rapid drug release and permeation kinetics alongside its high mucoadhesion.

## 1. Introduction

Nasal drug delivery has recently gained a lot of interest as a possible alternative drug administration route for numerous active substances. The main advantage of nasal administration includes the possibility of nose-to-brain drug delivery, where active substances can be absorbed through an axonal pathway via the olfactory and trigeminal nerves to the brain, bypassing the blood–brain barrier [1,2,3]. Other significant advantages include the highly vascularized nasal mucosa with a large surface area. Technological challenges arise from this administration route, mainly the rapid elimination of drugs from the nasal mucosa due to mucociliary clearance [4,5,6,7]. This elimination mechanism limits the residence time to below 20 min, requiring mucoadhesive excipients in nasal formulations to bypass this challenge. Another main feature is that the average administration volume is between 50 and 200 µL, thus requiring the active substances to be as concentrated as possible to increase the efficiency [8,9].

Solubilization techniques have been heavily investigated in current research and development landscapes for poorly water-soluble drugs, taking up most of the currently commercialized drugs [10,11]. Polymeric micelles are a novel nanocarrier system, made up from amphiphilic graft co-polymers via self-assembly, and are capable of enhancing the water solubility of numerous drugs [12,13]. Compared with classic surfactant materials, this nanoencapsulation technique offers higher drug loading and solubilization efficiency, stability, and the possibility of controlled drug release through various routes. Their average size ranges between 10 and 200 nm, suitable for rapid drug release and permeation [14,15]. However, this rapid release and diffusion profile also means that they can be washed away easily by mucociliary clearance due to the highly hydrophilic nature of the corona. As core-shell structures, they are heavily influenced by numerous excipients applied in nasal administration including hyaluronic acid, a gel-forming, mucoadhesive polymer [16,17].

Hyaluronic acid (HyA) is a linear polysaccharide that is made up of repeating disaccharide units of D-glucoronic acid and N-acetyl-D-glucosamine, linked by alternating β(1 → 3) and β(1 → 4) glycosidic bonds [18,19]. Hyaluronic acid can coat polymeric micelles whilst integrating inside the hydrophilic corona due to physicochemical bonds such as hydrogen bonding or van der Waals forces. This polysaccharide can form chemical bonds with mucin found in the nasal mucosa due to mucin’s sialic acid groups [20,21,22]. A coating of gel-like film is also formed on the nasal mucosa, which will increase the residence time. Despite its auspicious nature, different concentrations and forms can directly affect the nanoparticle characteristics of polymeric micelles. Polymeric micelles are widely applied for their rapid release and permeation nature; however, they can be controlled by numerous excipients hindering this property [5,16,23].

Nano spray-drying is a cutting-edge technology that allows for solid, more stable products compared with other polymeric micelle formulation techniques. In nasal and pulmonal delivery, its use is heavily favored due to the small particle size achievable via this drying technique. As stability is challenging for every nanoparticle formulation, many advantages may lie in the utilization of this drying technique. The production method also has limitations including that highly viscous liquids have poor yields and operation failures can occur [24,25,26].

During the study, vinpocetine was applied as a model active substance, indicated in neurodegenerative diseases, most specifically Alzheimer’s disease and in the case of stroke. It has poor water solubility, thus it is a suitable candidate for solubility enhancement via nanoencapsulation by polymeric micelles [27,28].

In this research article, the aim was to conduct base research and determine whether hyaluronic acid can be used to control the drug delivery of a polymeric micelle formulation characterized by rapid drug release and permeability profile. To find a balance between mucoadhesion and rapid drug release is a challenge, and thorough research must be conducted in order to find the optimal concentration. During our studies, the nano spray-dried particles were investigated in solid state and in liquid state to test whether they would fit the criteria for nanoparticles and nasal application.

## 2. Materials and Methods

### 2.1. Materials

Vinpocetine (VP, ethyl-apovincaminate) was applied as a model drug purchased from Sigma-Aldrich Co. Ltd. (Budapest, Hungary). As micelle forming agents, Soluplus^®^ (SP, poly(vinyl caprolactam)-poly(vinyl acetate)-poly(ethylene glycol) (PCL-PVAc-PEG)) was kindly gifted from BASF GmbH (Hannover, Germany), and Poloxamer 188 (P 188, poly(ethylene glycol)-block-poly(propylene glycol)-block-poly(ethylene glycol) (PEG-PPG-PEG)) was bought from Sigma-Aldrich Co. Ltd. As a bulk-forming agent, D-trehalose dihydrate (D-TRE); as mucoadhesive excipient, sodium hyaluronate (HyA, low molecular weight, 20–40 kDa); type III mucin and materials for simulated nasal electrolyte solution (SNES) were also bought from Sigma-Aldrich Co. Ltd. SNES was composed via the following: 8.77 g/L of sodium chloride, 0.59 g/L of anhydrous calcium chloride, and 2.98 g/L of potassium chloride in 1000 mL of purified water, and adjusted to a pH of 5.6 via 0.1 n hydrochloric acid [29]. Purified water was used for the experiments, which was filtered using a Millipore Milli-Q^®^ (Merck, Ltd., Budapest, Hungary) 140 Gradient Water Purification System.

### 2.2. Quantification of Vinpocetine via High-Performance Liquid Chromatography

The VP concentration was measured using high-performance liquid chromatography (HPLC) with an Agilent 1260 Infinity system (Agilent Technologies, Santa Clara, CA, USA). A Kinetex^®^ C18 column (5 µm, 150 mm × 4.6 mm, Phenomenex, Torrance, CA, USA) served as the stationary phase. Sample volumes of 10 µL were injected for analysis. The mobile phase consisted of a 1.54% *w*/*v* ammonium-acetate solution (A) and acetonitrile (B) in a 40:60 ratio. The separation was conducted using isocratic elution for 7 min at 40 °C, with a 1 mL/min flow rate. Detection was conducted at 280 ± 4 nm with a UV–Vis diode array detector. Chromatograms were analyzed using ChemStation B.04.03 software (Agilent Technologies, Santa Clara, CA, USA). The retention time for VP was 5.83 min, with a limit of detection (LOD) and limit of quantification (LOQ) of 6.31 ppm and 19.11 ppm, respectively. LOD and LOQ values were determined according to the ICH Q2(R2) guideline [30]. Calibration was performed over a range of 20 to 100 µg/mL, yielding a coefficient of linearity (R^2^) of 0.9997.

### 2.3. Formulation of Polymeric Micelles via Nano Spray-Drying

At first, various concentrations of HyA were dissolved in cold (4 °C) purified water in the concentration of 0–0.5% *w*/*v* for a total volume of 25 mL each. Based on preliminary results, 300 mg SP and 250 mg P 188 were dissolved in each HyA solution. Separately, 25 mL of 1 mg/mL of vinpocetine ethanolic solution was prepared. The two solutions were mixed together for 4 h at ambient temperature on a magnetic stirrer (750 rpm). Finally, 5.0 g of D-TRE was dissolved in the mixture.

To prepare the samples, a Büchi Nano Spray Dryer (Büchi Nano Spray Dryer B-90 HP, Büchi, Flawil, Switzerland) was used that was equipped with a small nebulizer. Based on prior experiments, the following settings were utilized: aspirator capacity—100%; airflow rate—115 mL/min; inlet temperature—100 °C; and a pump rate of 20%. The yield was measured as the percentage of the collected powder against the measured dry amount, which ranged from 75.41 to 82.03%. For the liquid state measurements, purified water was used as a solvent, where the target concentration of VP was 250 µg/mL. This concentration was checked via HPLC and adjusted before each measurement.

### 2.4. Characterization of the Nano Spray-Dried Powders

#### 2.4.1. Characterization of Morphology via Scanning Electron Microscopy

The morphology of the nano spray-dried formulations was analyzed using scanning electron microscopy (SEM) (Hitachi S4700, Hitachi Scientific Ltd., Tokyo, Japan). An air pressure ranging from 1.3 to 13.1 mPa was applied, with a high voltage of 10 kV and a current of 10 mA. To make the samples conductive, they were sputter-coated with a gold-palladium alloy using a high-vacuum evaporator under an argon atmosphere (Bio-Rad SC 502, VG Microtech, Uckfield, UK). The gold-palladium coating was applied to a thickness of 10 nm.

#### 2.4.2. Determination of Particle Size via Laser Diffraction

Laser diffraction was employed to determine the particle size and particle size distribution (expressed as Span) of the nano spray-dried formulations (Malvern Mastersizer Scirocco 2000, Malvern Instruments Ltd., Worcestershire, UK) using a dry dispersion unit. Approximately 0.1–0.3 g of the sample was placed in the feeding tray. The dispersion unit was set to 3.0 bar, and a vibration feed of 75% was applied. All measurements were carried out in triplicate with individual batches (*n* = 3), and the results are expressed as the average ± SD.

#### 2.4.3. Characterization of Crystallinity via X-Ray Powder Diffraction

The crystalline structure of the nano spray-dried products was analyzed using X-ray powder diffraction (XRPD) with a Bruker D8 Advance X-ray diffractometer (Bruker AXS GmbH, Karlsruhe, Germany) employing Cu Kα radiation (λ = 1.5406 Å) and a VANTEC-1 detector (Bruker AXS GmbH, Karlsruhe, Germany). The measurements were conducted at 40 kV and 40 mA. The angular range for the analysis was set from 3° to 40° 2θ, with a step time of 0.1 s and a step size of 0.007°. Data manipulation and evaluation were performed using DIFFRAC.EVA software v6 (Bruker AXS GmbH, Karlsruhe, Germany).

### 2.5. Characterization of the Polymeric Micelles in Liquid State

#### 2.5.1. Determination of Micelle Size, Size Distribution, and Zeta Potential

To determine the micelle size (expressed as the average hydrodynamic diameter (D_H_)), the micelle size distribution (expressed as the polydispersity index, PdI), and the zeta potential (ζ), dynamic light scattering (DLS) measurements were applied using a Malvern Nano ZS Zetasizer (Malvern Instruments, Worcestershire, UK). Zeta potential was measured based on the Smoluchowski model. Samples were measured in folded capillary cells at 25 °C, with a refractive index of 1.650. All measurements were carried out in triplicate with individual batches (*n* = 3), and the results are expressed as the average ± SD.

#### 2.5.2. Determination of Encapsulation Efficiency

For the determination of encapsulation efficiency (EE), samples were dissolved at the target VP concentration of 250 µg/mL and placed in Spin-X^®^ centrifuge tubes (Costar, Salt Lake City, UT, USA) [31]. The micelles were filtered through a cellulose acetate membrane filter with a 0.22 µm cut-off pore diameter inside the polypropylene tube via centrifugation using a Hermle Z323 K high-performance refrigerated centrifuge (Hermle AG, Gosheim, Germany). The separation was performed at 13,500 rpm and 4 °C for 45 min, followed by quantitative measurements via HPLC. All measurements were carried out in triplicate with individual batches (*n* = 3), and the results expressed as the average ± SD. EE was calculated via the following equation:

(1) $$\mathrm{EE}\left(\%\right)=\frac{\mathrm{measured}\;\mathrm{VP}\;\left(\mathrm{mg}\right)}{\mathrm{initial}\;\mathrm{VP}\;\left(\mathrm{mg}\right)}\times 100$$

#### 2.5.3. Determination of Thermodynamic Solubility

Quantitative determination of the solubility enhancement was performed via the measurement of thermodynamic solubility with the saturation method [32]. A total of 0.5 mL of purified water was measured into vials, and the formulations were dissolved until visible saturation (i.e., excess particles were visible in the liquid). The samples were covered with parafilm and constantly kept under stirring for 72 h at ambient temperature and a stirring rate of 100 rpm. Solutions were filtered through a 0.22 µm pore-sized polyether sulfone membrane. The passed-through concentration was measured via HPLC. All measurements were carried out in triplicate with individual batches (*n* = 3), and the results expressed as the average ± SD.

#### 2.5.4. Determination of Viscosity

The viscosity of the dispersed formulations was evaluated using a Physica MCR 302 rheometer (Anton Paar, Graz, Austria). A cone-and-plate measuring system was employed, featuring a 25 mm diameter, a 1° cone angle, and a 0.05 mm gap height at the center of the cone. Flow curves for the samples were generated over a shear rate range of 0.1 to 100 s^−1^. The viscosity of the samples was determined at a shear rate of 50 s^−1^ through the interpolation function provided by the RheoCompass software v1.35.1394 (Anton Paar GmbH, Ashland, VA, USA). All measurements were carried out in triplicate with individual batches (*n* = 3), and the results expressed as the average ± SD.

### 2.6. Nasal Applicability Studies

#### 2.6.1. In Vitro Mucoadhesion Study

To evaluate the mucoadhesive nature of the formulations, tensile tests were conducted using a TA-XT Plus Texture Analyzer (Metron Ltd., Budapest, Hungary) equipped with a 5 kg load cell and a 1 cm diameter cylinder probe. The dispersed formulations were brought into contact with a 25 mm diameter filter paper disc moistened with 50 µL of 8% w/w porcine mucin (type III) dispersion in SNES. The 20 µL samples were applied to the filter paper, which was secured to the cylinder probe and placed into contact with the mucin dispersion. A preload of 2500 mN was applied for 3 min, after which the cylinder probe was moved upward at a set speed of 2.5 mm/min to separate the contact surfaces. The mucoadhesivity was assessed using the adhesive force (F, mN) and adhesive work (A, mN × mm).

#### 2.6.2. In Vitro Drug Release Study

The drug release profile was investigated via the paddle dissolution method, where dispersed formulations were placed in dialysis tubes (Spectra/Por^®^ Dialysis Membrane with a 12–14 kDa MWCO (Spectrum Laboratories Inch., Rancho Dominguez, CA, USA)). As dissolution media, 100 mL of SNES was used to ensure sink conditions. Measurements were performed at 32 °C under 100 rpm of paddle rotation [33]. Aliquots were taken at predetermined time points up to 60 min, followed by a quantification of vinpocetine by HPLC. Three parallel measurements were performed from individual batches and the results expressed as the means ± SD.

#### 2.6.3. In Vitro Drug Permeation Study

The in vitro nasal permeation study was performed in a modified Side-bi-Side^®^ type horizontal diffusion cell, where a cellulose membrane impregnated with isopropyl myristate was used as a diffusion barrier. The surface area of the membrane was 0.785 cm^2^. The donor compartment consisted of 9.0 mL of each formulation dissolved in SNES, whilst the acceptor compartment was 9.0 mL of pH 7.4 PBS. Aliquots were taken at predetermined time points followed by the quantification of VP via HPLC. Cumulative permeability was calculated from the mass permeated divided by the surface area of the membrane. Three parallel measurements were performed from individual batches and the results expressed as the means ± SD.

### 2.7. Stability Studies

Stability studies were conducted according to the ICH Q1A(R2) guideline, where the spray-dried particles were kept at 25 °C and 60% relative humidity for 6 months [34]. Samples were measured monthly via laser diffraction to characterize the particle size in solid state and via dynamic light scattering to determine the changes in the colloidal solution state.

## 3. Results

### 3.1. Characterization of the Nano Spray-Dried Powders

#### 3.1.1. Determination of Particle Size and Size Distribution

The particle size of the nano spray-dried samples was investigated via laser diffraction to describe the effect of various hyaluronic acid concentrations on the particles. The results in Table 1 show that the mean particle size (D[0.5]) decreased whilst increasing the concentration of HyA up to 0.4% *w*/*v*. Above this, at the highest concentration, the particles had a significantly higher (**, *p* < 0.01) particle size, which indicated particle aggregation. The high concentration of HyA can also contribute to the increase in the adhesiveness between the particles, as intermolecular interactions, such as hydrogen bonding, can also lead to aggregated particles, thus they are not dispersed to the extent of the others [35]. The increased viscosity in the feed solution can also lead to the formation of larger droplets during atomization, resulting in larger dried particles. The Span value also followed the same tendency, corroborating the particle sizes whilst indicating a monodisperse size distribution. The yield of each formulation was between 71.2 and 84.5%, whilst at a 0.5% *w*/*v* HyA concentration, this value dropped to 53.2%, which was not satisfactory.

#### 3.1.2. Characterization of Morphology

**Figure 1:**
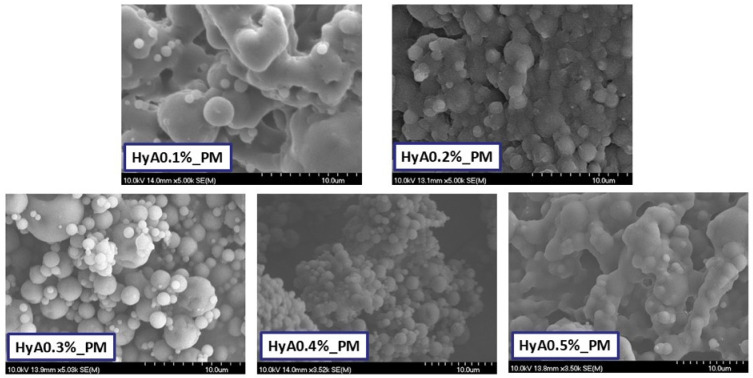
Scanning electron microscopic images of the polymeric micelle formulation at various hyaluronic acid concentrations. The unit of scale is 10 µm.

The morphology of the formulations was characterized via scanning electron microscopy (SEM). The captured images can be seen in Figure 1. Reflecting on the results of the laser-diffraction based particle size determination and findings, the main difference between the results lay in the mechanism of the specific measurement, as in the SEM images, the particles were not dispersed, thus providing a better quality of particle size determination. In the case of the lower and highest concentrations of HyA (0.1–0.2; 0.5% *w*/*v*), the presence of smaller spherical particles on the surfaces of larger ones suggests either agglomeration or coalescence, which can be due to the adhesive nature of hyaluronic acid. The particles were irregularly shaped with smooth surfaces. At 0.3% *w*/*v*, the particles took on a spherical shape, but the particle size distribution was high based on the image. At a 0.4% *w*/*v* HyA concentration, the particles were uniformly shaped and had a spherical morphology with smooth edges and surface. Grouping of particles could be found as agglomerations. Larger particles could be also found, but the size difference of various particles was not as comparable as for at the other formulations.

Further analysis was conducted, where the average particle size of 50 particles was investigated based on the SEM captures (Table 2). The results followed a similar tendency to the measurements of the laser diffraction study. Particles seemed a bit larger, however, the reason behind this lies in the different analytical techniques; as in the case of laser diffraction, the particles were in a more dispersed state, not clumped together like in the SEM images.

#### 3.1.3. Characterization of Crystallinity via X-Ray Powder Diffraction

**Figure 2:**
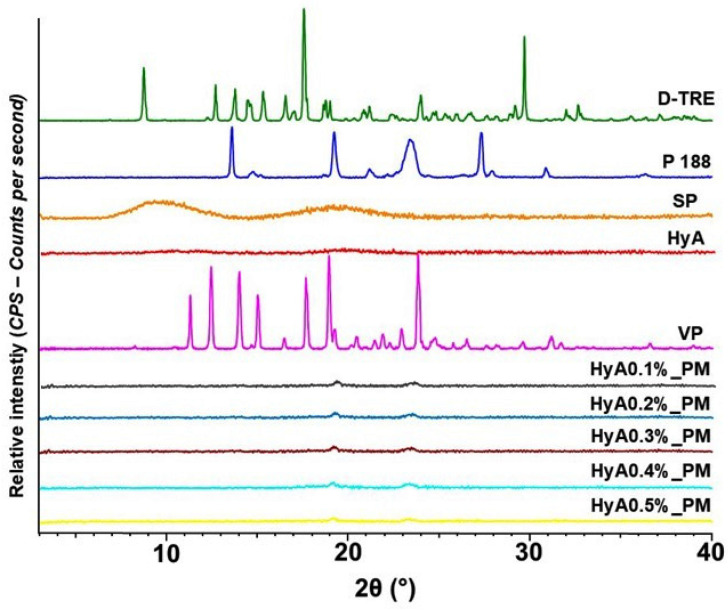
Diffractograms of the nano spray-dried products (HyA0.1–0.5%_PM) and the initial polymeric-micelle forming components and the active substance, vinpocetine (VP).

X-ray powder diffraction was used to analyze the crystallinity of the products and initial materials. The diffractograms of each material and the spray-dried products are shown in Figure 2.

Characteristic crystalline peaks could be found in the diffractogram of vinpocetine that could not be found in the diffractograms of each spray-dried formulation. D-trehalose, also characterized by sharp crystalline peaks, also faded in the formulation, explained by the amorphization via spray drying. The applied polymers also had an amorphous nature as no crystalline peaks could be detected. Besides the temperature and size reduction-mediated amorphization, polymers also play a crucial role in inhibiting crystallization through steric hindrance. The hydroxyl groups of the applied co-polymers can also stabilize the amorphous structure by hydrogen bond formation or through van der Waals interactions. At the moment of drying, the overall glass transition temperature of the drugs can also increase, leading to a reduced molecular stability, allowing for a stable amorphous structure after drying.

### 3.2. Characterization of the Polymeric Micelles in a Liquid State

The higher surface area and the higher free energy state due to the amorphous nature led to rapid dispersion in aqueous media, ranging between 15 and 40 s, increasing gradually with the hyaluronic acid concentration. As critical factors, micelle size and size distribution were first measured by dynamic light scattering, the results of which are shown in Table 3.

The concentration of hyaluronic acid directly affects the micelle size, which can be due to various reasons. Hyaluronic acid can form hydrogen bonds between the co-polymers applied for micelle formulation, which can lead to polymer chain stretching via the conformation change. Therefore, the hydrophilic corona may increase in size, leading to a less dense core—shell structure. Formulations with hyaluronic acid contents ranging from 0.1 to 0.4% *w*/*v* could all be accepted in their typical micelle size range as suitable carriers for nasal administration, whilst at the highest concentration, not only the size, but also the distribution was increased. The optimal nano-sized characteristics can be described in the formulation of 0.4% *w*/*v* HyA concentration. Its size distribution was monodispersed as the polydispersity index was below 0.300, a characteristic that could not be found in the other formulations. Monodisperse size distribution is of paramount importance due to uniformity in loading, leading to a uniform drug release and permeability profile. Accurate dosing can be also mentioned here as well as manufacturing consistency. Whilst polymeric micelles are considered as non-biological complex drugs (NBCDs) from a regulatory viewpoint, it is still a challenging area that can be aided by proper formulation control.

The zeta potential of the formulations also increased gradually with the increase in HyA concentration. This helped to reduce particle clumping due to electrostatic repulsion between the particles. At lower zeta potential values, nanoparticles may aggregate due to van der Waals forces, increasing their size distribution and making them less uniform to dissolve or permeate through biological barriers. Negatively charged micelles also tend to permeate via paracellular transport through mucosal barriers or via transcytosis.

To characterize the solubilization via polymeric micelle formation, the encapsulation efficiency and the thermodynamic solubility were determined. The results of these measurements are summarized in Table 4.

Hyaluronic acid, as a water-soluble polymer, can increase the overall hydrophilicity of polymeric micelles via its incorporation into the hydrophilic corona. The incorporation can enhance the hydration of the shell or lead to swelling of the polymeric micelles whilst stabilizing the core-shell structure. HyA can also influence micelle stability via the reduction in drug leakage, as drug–carrier interactions are overall increased due to electrostatic interactions or non-covalent binding. Due to similar or the same reasons, hyaluronic acid can also positively influence the thermodynamic solubility of the encapsulated drug. HyA can form a hydrated outer shell around the core-shell structure, aiding to solvate the poorly water-soluble encapsulated drugs by providing a higher degree of thermodynamic stability. Hyaluronic acid also has a slight sensitivity to pH due to its carboxyl groups. In slightly acidic conditions, which is typical for nasal fluid (the pH is approximately 5.5–6.5), the ionization state changes, altering the structure of polymeric micelles, which could also increase the stability and prevent drug leaking [5,36,37]. The results presented in Table 3 demonstrate that the encapsulation efficiency, as the drug encapsulated in the micellar core, increased with the HyA concentration, but at the highest concentration, this tendency broke down. This corroborates the results of nanoparticle characterization, as the micelles had a higher size distribution and size at this concentration. All formulations significantly increased the solubility of vinpocetine (*** *p* < 0.001), gradually increasing to that similar to the encapsulation efficiency values.

**Figure 3:**
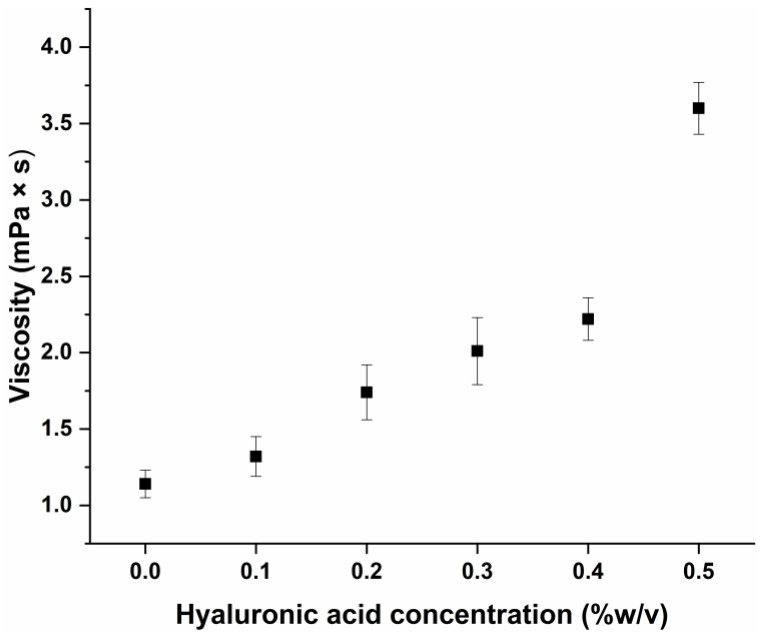
Viscosity of hyaluronic acid-modified polymeric micelles loaded with polymeric micelles. Data are presented as the average ± SD (*n* = 3).

The viscosity of the formulations was also investigated to test the theory in order to develop a value-added formulation with low viscosity with high mucoadhesive properties to enhance rapid drug release and permeation whilst increasing the residence time on the nasal mucosa. Too high a viscosity can lead to hindered drug release, as the higher viscosity creates a diffusion barrier to the movement of drug molecules. Polymeric micelles are also characterized by the high rate of Fickian diffusion, which can be resisted by viscous systems. The viscosity of the formulation can be seen in Figure 3.

### 3.3. In Vitro Nasal Applicability Studies

To validate the formulations and compare their individual properties on nasal administration, in vitro mucoadhesion, release, and permeation studies were performed.

**Figure 4:**
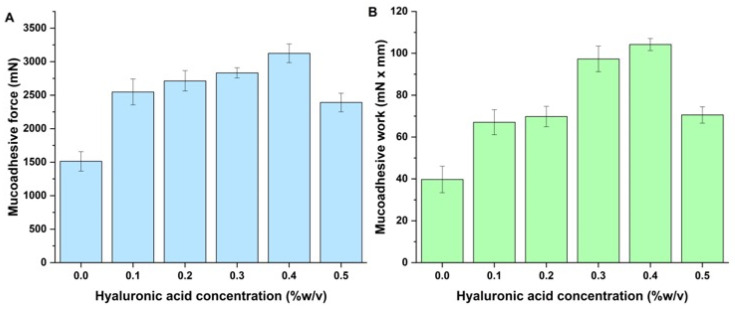
(**A**) Mucoadhesive force and (**B**) mucoadhesive work of the hyaluronic acid-modified polymeric micelles and the hyaluronic acid-free polymeric micelle formulation.

Hyaluronic acid is a polyanionic polysaccharide with a high carboxyl group ratio. Therefore, it can form electrostatic interactions and hydrogen bonds with the mucin glycoproteins found in the mucus layer. Even though the mucus is also negatively charged, sialic acid residues can form strong mucoadhesion with HyA. Hyaluronic acid can also interact with cell surface receptors, like CD44, which would also help the uptake of drugs [38,39]. Hyaluronic acid can also form viscous gels at higher concentrations, which would also benefit locally, as HyA can form a gel-like film on the mucosal surface. The results of the in vitro mucoadhesion study are demonstrated in Figure 4.

Compared with the HyA-free polymeric micelles, all formulations had naturally higher mucoadhesive properties due to the presence of hyaluronic acid. A slight increase in the tendency could also be seen with the increase in HyA, which was reduced at the highest concentration of HyA. This can be explained by the decrease in polymer chain mobility, which is required for interpenetration and hydrogen bonding with mucin. The dense gel-like matrix does not have a good spreading property to ensure proper wetting of the mucosal surface. The steric hindrance at high concentrations also hinders the adhesive force between the nasal mucosa and the polymer chain matrix.

**Figure 5:**
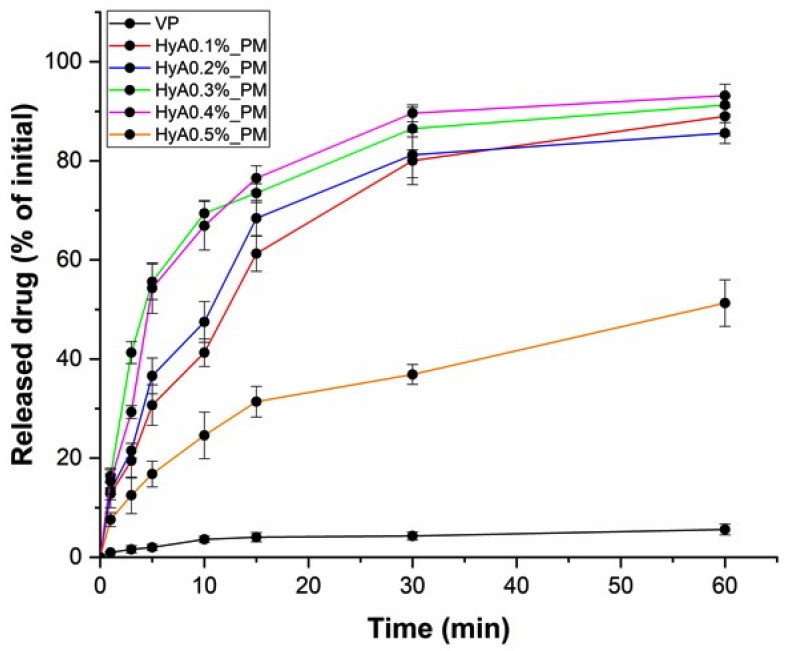
In vitro drug release study performed in the simulated nasal electrolyte solution. Results are expressed as the percentage of the released amount compared with the initial amounts. Results are presented as the average ± SD (*n* = 3).

In vitro drug release studies were carried out to compare the kinetic profile of the individual formulations compared with the initial vinpocetine. The aim was to describe the effect of HyA on the release profile whilst finding the one(s) that would fulfill the criteria of a rapid drug release system along with high mucoadhesion. The drug release test results are shown in Figure 5.

As a reference, a vinpocetine quasi-suspension was used that had a limited water solubility, resulting in poor drug release over the investigated period. All formulations had a significantly higher (HyA0.5% vs. VP, ** *p* < 0.01; HyA0.1–0.4% vs. VP, *** *p* < 0.001) drug release profile. The formulations did not differ from each other significantly, except for the one with the highest concentration of HyA (HyA0.1–0.4% vs. HyA0.5%, ** *p* < 0.01). This can be explained by the steric hindrance of the hyaluronic chains packed closely together, which formed a dense diffusion resistance to the embedded polymeric micelles and the drug itself. Analyzing the results proved that our goal was successful, as approximately 70–80% of the drug was released by 15 min, a critical time based on mucociliary clearance. The results were also corroborated with the small particle size and uniform distribution-mediated solubility enhancement.

**Figure 6:**
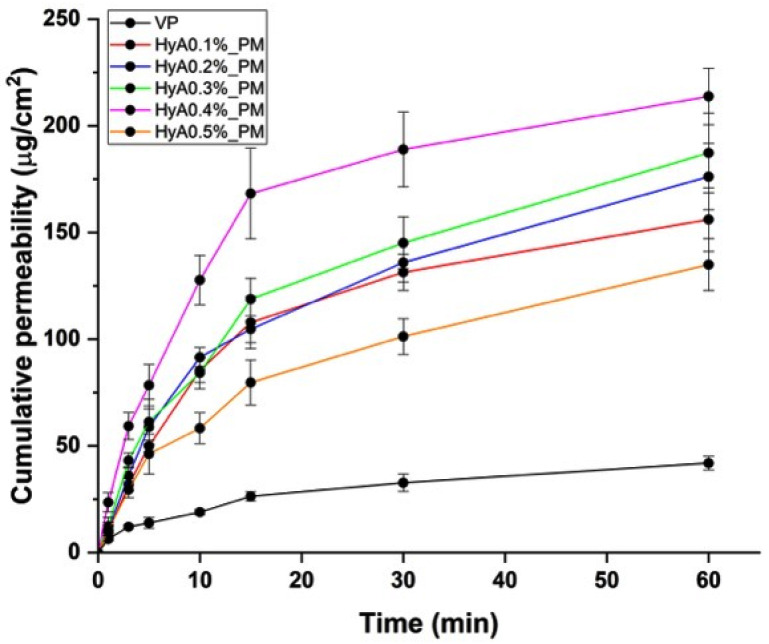
Results of the in vitro permeability study. Data are represented as the cumulative permeability (as the mass permeated through a unit of surface area) versus the time. Data are presented as the average ± SD (*n* = 3).

In vitro drug permeation studies were also performed to test whether the hyaluronic acid concentration could be optimized to ensure high and rapid drug permeability whilst offering the possibility of higher mucoadhesion. The results of the in vitro permeability study are demonstrated in Figure 6.

The results were similar to the in vitro drug release data, however, there was not much of a difference between the different concentrations of HyA added to the formulations. The best performance was shown in the case of HyA0.4%_PM, with the highest permeated drug mass through the surface. Even though hyaluronic acid can theoretically hinder drug release, it also enhances the drug permeation. Hyaluronic acid has a hydrating property that would loosen the tight junctions between epithelial cells, leading to enhanced paracellular transport.

### 3.4. Stability Studies

**Figure 7:**
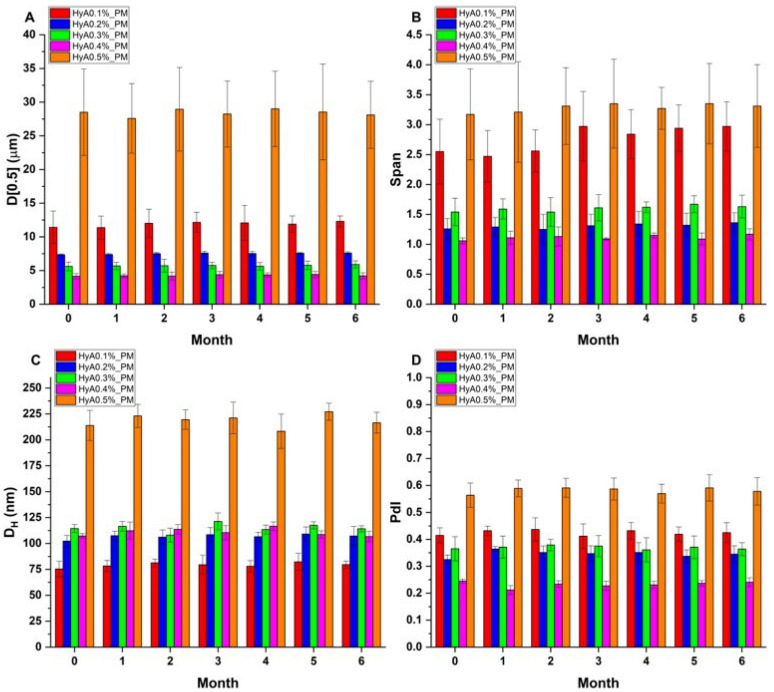
Stability test of the polymeric micelle formulations at 25 ± 2 °C/60 ± 5% RH according to ICH Q1A(R2). Laser diffraction and dynamic light scattering measurements were conducted monthly. (**A**) Average particle size (D[0.5]); (**B**) particle size distribution (Span); (**C**) average hydrodynamic diameter (D_H_); (**D**) polydispersity index (PdI). Data are presented as the average ± SD (*n* = 3).

Stability studies were conducted during a 6-month period to ensure that the formulation would perform over the long-term. Nano spray-dried formulations were kept at 25 ± 2 °C/60 ± 5% relative humidity (RH) values in a chamber according to the ICH Q1A(R2) guideline. Each month, laser diffraction measurements were carried out to test the stability of the solid products, which were then dissolved in purified water and the micelle characteristics measured via dynamic light scattering. The formulations remained stable during the measurement period, and no significant changes were observed. The results are summarized in Figure 7.

## 4. Discussion

Based on the results, one suitable candidate was chosen for further evaluation as its features met the criteria at multiple measurements. The formulation with 0.4% *w*/*v* fulfilled the set goal, as it acted as a rapid drug release and permeability system with high mucoadhesive tendencies. The nano spray-dried particle had an average particle size of 4.15 ± 0.41 µm with a span value of 1.06 ± 0.05. This small particle size and monodisperse size distribution allowed for rapid dissolution in aqueous media and was proven to be stable for 6 months at ambient conditions. It also had the highest yield amongst the others (approximately 84.5%), proving that nano spray-drying is a suitable formulation technique to develop hyaluronic acid-modified polymeric micelles. Generally, polymeric micelles are spray-dried for inhalation purposes due to the favorable particle size ranging from 1 to 5 µm [40]. Lipid particles tended to have a smaller particle size upon spray-drying compared with our results, however, the bulk of trehalose provided an excellent physical stability for a longer period of time [41]. Hyaluronic acid can also increase the particle size due to the previously mentioned reasons, namely that at higher concentrations, it promotes particle aggregation due to the high adhesive nature of this polymer [42,43]. Its amorphous nature also helps with the dispersion to act as an ex tempore dispersible solid state dosage form.

Its nanoparticle characteristics were also preferable, with a micelle size of 107.3 ± 2.1 nm in monodisperse distribution and high colloidal stability with a zeta potential of 34.5 ± 2.5 mV. The negative surface charge also promoted paracellular transport across the nasal mucosa, which is favorable in the case of nanoparticles as they would not be hindered through the negatively-charged cells via transcellular absorption. The solubilization was successful with a high encapsulation efficiency above 89% and a significant increase in thermodynamic solubility in aqueous media.

These properties led to an efficient drug release and permeation, where the active substance was released above 70% in 15 min with a rapid and high rate of permeation. This was corroborated by the colloidal properties of the formulation, where the small micelle size and high solubilization efficiency mediated the rapid release kinetics. Meanwhile, the formulation had low viscosity with high mucoadhesion, a prosperous combination of nasal liquid dosage forms, as it would help to adhere to the nasal mucosa whilst not hindering drug diffusion from the polymeric matrices. In the literature, it can be found that hyaluronic acid is usually applied for nasal administration to facilitate the delivery of antigens [23] and can also enhance the drug delivery of small molecular-weight drugs, especially for the auspicious nose-to-brain delivery. In a similar work, sodium hyaluronate was applied to facilitate the drug transport to the brain, with similar release profiles to our work [44].

## 5. Conclusions

In conclusion, it can be claimed that the base research proved useful in finding a potential candidate polymeric micelle formulation coated by hyaluronic acid. The value-added formulation has the potential to deliver this drug to a high extent across the nasal mucosa whilst having a high mucoadhesive nature. The sprayable, low viscosity formulation provided a rapid drug release profile for the optimal sample, further facilitating nasal absorption.

## Figures and Tables

**Table 1:** Effect of hyaluronic acid on the size of the nano spray-dried particles. Data are presented as the means ± SD (*n* = 3).

HyA Concentration (% *w*/*v*)	0.1	0.2	0.3	0.4	0.5
D[0.5] (µm)	11.41 ± 2.40	7.34 ± 0.91	5.62 ± 1.64	4.15 ± 0.41	28.51 ± 6.41
Span	2.55 ± 0.54	2.26 ± 0.17	1.54 ± 0.23	1.06 ± 0.05	3.17 ± 0.76
Yield (%)	75.3 ± 2.4	71.2 ± 4.1	80.4 ± 3.9	84.5 ± 2.2	53.2 ± 2.7

**Table 2:** Average particle size of the spray-dried formulations based on the scanning electron microscopic images. Data are presented as the means ± SD (*n* = 50, as 50 particles were investigated).

HyA Concentration (% *w*/*v*)	0.1	0.2	0.3	0.4	0.5
D[0.5] (µm)	14.27 ± 3.12	6.98 ± 1.24	6.11 ± 2.49	4.87 ± 0.89	36.75 ± 7.74

**Table 3:** Micelle size (D_H_), size distribution (PdI), and zeta potential (ζ) of hyaluronic-acid coated, vinpocetine-loaded polymeric micelles measured via dynamic light scattering. Data are presented as the means ± SD (*n* = 3).

HyA Concentration (% *w*/*v*)	0.1	0.2	0.3	0.4	0.5
D_H_ (nm)	75.4 ± 7.4	102.3 ± 5.4	114.5 ± 3.7	107.3 ± 2.1	213.8 ± 14.5
PdI	0.415 ± 0.028	0.325 ± 0.017	0.365 ± 0.045	0.245 ± 0.007	0.564 ± 0.045
ζ (mV)	−15.3 ± 2.4	−23.1 ± 3.7	−27.5 ± 1.5	−34.5 ± 2.5	−35.8 ± 6.5

**Table 4:** Encapsulation efficiency (EE%) and thermodynamic solubility (S) of the polymeric micelle formulations based on the added hyaluronic acid compared with the initial vinpocetine. Data are presented as the means ± SD (*n* = 3).

HyA Concentration (% *w*/*v*)	VP (0)	0.1	0.2	0.3	0.4	0.5
EE%	-	76.5 ± 4.5	81.2 ± 3.1	84.5 ± 2.6	89.5 ± 3.7	75.4 ± 6.7
S_25°C_ (µg/mL)	2.39 ± 0.37	564.4 ± 31.2	607.7 ± 24.6	687 ± 19.6	769.4 ± 24.2	649.1 ± 38.1

## Data Availability

The data presented in this study are available upon request from the corresponding author.

## Abbreviations

The following abbreviations are used in this manuscript:

D[0.5]	Average particle size
D_H_	Average hydrodynamic diameter, micelle size
DLS	Dynamic light scattering
D-TRE	D-trehalose dihydrate
EE	Encapsulation efficiency
HPLC	High-performance liquid chromatography
HyA	Hyaluronic acid
ICH	International Council for Harmonization of Technical Requirements for Pharmaceuticals for Human Use
LOD	Limit of detection
LOQ	Limit of quantification
P 188	Poloxamer 188
PBS	Phosphate buffered saline
PCL-PVAc-PEG	Poly(vinyl caprolactam)-poly(vinyl acetate)-poly(ethylene glycol)
PdI	Polydispersity index
PEG-PPG-PEG	Poly(ethylene glycol)-poly(propylene glycol)-poly(ethylene glycol)
RH	Relative humidity
SEM	Scanning electron microscopy
SNES	Simulated nasal electrolyte solution
SP	Soluplus
VP	Vinpocetine
XRPD	X-ray powder diffraction
ζ	Zeta potential

## References

[1] Wang Z., Xiong G., Tsang W.C., Schätzlein A.G., Uchegbu I.F. (2019). Nose-to-Brain Delivery. J. Pharmacol. Exp. Ther..

[2] Chavda V.P., Jogi G., Shah N., Athalye M.N., Bamaniya N., Vora L.K., Cláudia Paiva-Santos A. (2022). Advanced Particulate Carrier-Mediated Technologies for Nasal Drug Delivery. J. Drug Deliv. Sci. Technol..

[3] Williams G., Suman J.D. (2022). In Vitro Anatomical Models for Nasal Drug Delivery. Pharmaceutics.

[4] Rabiee N., Ahmadi S., Afshari R., Khalaji S., Rabiee M., Bagherzadeh M., Fatahi Y., Dinarvand R., Tahriri M., Tayebi L. (2021). Polymeric Nanoparticles for Nasal Drug Delivery to the Brain: Relevance to Alzheimer’s Disease. Adv. Ther..

[5] Laffleur F., Bauer B. (2021). Progress in Nasal Drug Delivery Systems. Int. J. Pharm..

[6] Gholizadeh H., Ong H.X., Bradbury P., Kourmatzis A., Traini D., Young P., Li M., Cheng S. (2021). Real-Time Quantitative Monitoring of in Vitro Nasal Drug Delivery by a Nasal Epithelial Mucosa-on-a-Chip Model. Expert Opin. Drug Deliv..

[7] Rai G., Gauba P., Dang S. (2023). Recent Advances in Nanotechnology for Intra-Nasal Drug Delivery and Clinical Applications. J. Drug Deliv. Sci. Technol..

[8] Huang C.-W., Chuang C.-P., Chen Y.-J., Wang H.-Y., Lin J.-J., Huang C.-Y., Wei K.-C., Huang F.-T. (2021). Integrin A2β1-Targeting Ferritin Nanocarrier Traverses the Blood–Brain Barrier for Effective Glioma Chemotherapy. J. Nanobiotechnol..

[9] Kaur J., Gulati M., Kapoor B., Jha N.K., Gupta P.K., Gupta G., Chellappan D.K., Devkota H.P., Prasher P., Ansari M.S. (2022). Advances in Designing of Polymeric Micelles for Biomedical Application in Brain Related Diseases. Chem.-Biol. Interact..

[10] Rajput A., Pingale P., Dhapte-Pawar V. (2022). Nasal Delivery of Neurotherapeutics via Nanocarriers: Facets, Aspects, and Prospects. Front. Pharmacol..

[11] Sastri K.T., Gupta N.V., M S., Chakraborty S., Kumar H., Chand P., Balamuralidhara V., Gowda D.V. (2022). Nanocarrier Facilitated Drug Delivery to the Brain through Intranasal Route: A Promising Approach to Transcend Bio-Obstacles and Alleviate Neurodegenerative Conditions. J. Drug Deliv. Sci. Technol..

[12] Abo El-Enin H.A., Ahmed M.F., Naguib I.A., El-Far S.W., Ghoneim M.M., Alsalahat I., Abdel-Bar H.M. (2022). Utilization of Polymeric Micelles as a Lucrative Platform for Efficient Brain Deposition of Olanzapine as an Antischizophrenic Drug via Intranasal Delivery. Pharmaceuticals.

[13] Dong J., Wang Y., Zhang J., Zhan X., Zhu S., Yang H., Wang G. (2013). Multiple Stimuli-Responsive Polymeric Micelles for Controlled Release. Soft Matter.

[14] Kotta S., Aldawsari H.M., Badr-Eldin S.M., Nair A.B., Yt K. (2022). Progress in Polymeric Micelles for Drug Delivery Applications. Pharmaceutics.

[15] Perumal S., Atchudan R., Lee W. (2022). A Review of Polymeric Micelles and Their Applications. Polymers.

[16] Li M., Zhang L., Xuan Y., Zhi D., Wang W., Zhang W., Zhao Y., Zhang S., Zhang S. (2022). pH-Sensitive Hyaluronic Acid-Targeted Prodrug Micelles Constructed via a One-Step Reaction for Enhanced Chemotherapy. Int. J. Biol. Macromol..

[17] Niu J., Yuan M., Zhang Z., Wang L., Fan Y., Liu X., Liu X., Ya H., Zhang Y., Xu Y. (2022). Hyaluronic Acid Micelles for Promoting the Skin Permeation and Deposition of Curcumin. Int. J. Nanomed..

[18] Yasin A., Ren Y., Li J., Sheng Y., Cao C., Zhang K. (2022). Advances in Hyaluronic Acid for Biomedical Applications. Front. Bioeng. Biotechnol..

[19] Abatangelo G., Vindigni V., Avruscio G., Pandis L., Brun P. (2020). Hyaluronic Acid: Redefining Its Role. Cells.

[20] Bayer I.S. (2020). Hyaluronic Acid and Controlled Release: A Review. Molecules.

[21] Gupta R.C., Lall R., Srivastava A., Sinha A. (2019). Hyaluronic Acid: Molecular Mechanisms and Therapeutic Trajectory. Front. Vet. Sci..

[22] Marinho A., Nunes C., Reis S. (2021). Hyaluronic Acid: A Key Ingredient in the Therapy of Inflammation. Biomolecules.

[23] Suzuki K., Yoshizaki Y., Horii K., Murase N., Kuzuya A., Ohya Y. (2022). Preparation of Hyaluronic Acid-Coated Polymeric Micelles for Nasal Vaccine Delivery. Biomater. Sci..

[24] Chopde S., Datir R., Deshmukh G., Dhotre A., Patil M. (2020). Nanoparticle Formation by Nanospray Drying & Its Application in Nanoencapsulation of Food Bioactive Ingredients. J. Agric. Food Res..

[25] Jayaprakash P., Maudhuit A., Gaiani C., Desobry S. (2023). Encapsulation of Bioactive Compounds Using Competitive Emerging Techniques: Electrospraying, Nano Spray Drying, and Electrostatic Spray Drying. J. Food Eng..

[26] Arpagaus C. (2019). PLA/PLGA Nanoparticles Prepared by Nano Spray Drying. J. Pharm. Investig..

[27] Panda P.K., Ramachandran A., Panda P., Sharawat I.K. (2022). Safety and Efficacy of Vinpocetine as a Neuroprotective Agent in Acute Ischemic Stroke: A Systematic Review and Meta-Analysis. Neurocrit. Care.

[28] Al-Kuraishy H., Al-Gareeb A., Naji M., Al-Mamorry F. (2020). Role of Vinpocetine in Ischemic Stroke and Poststroke Outcomes: A Critical Review. Brain Circ..

[29] Sipos B., Csóka I., Budai-Szűcs M., Kozma G., Berkesi D., Kónya Z., Balogh G.T., Katona G. (2021). Development of Dexamethasone-Loaded Mixed Polymeric Micelles for Nasal Delivery. Eur. J. Pharm. Sci..

[30] International Council for Harmonisation of Technical Requirements for Pharmaceuticals for Human Use (ICH) (2022). ICH Harmonised Guideline: Q2(R2)—Validation of Analytical Procedures. https://www.Ich.Org/Page/Quality-Guidelines.

[31] Katona G., Sipos B., Ambrus R., Csóka I., Szabó-Révész P. (2022). Characterizing the Drug-Release Enhancement Effect of Surfactants on Megestrol-Acetate-Loaded Granules. Pharmaceuticals.

[32] Sipos B., Katona G., Szarvas F.M., Budai-Szűcs M., Ambrus R., Csóka I. (2023). Development of Vinpocetine-Loaded Nasal Polymeric Micelles via Nano-Spray-Drying. Pharmaceuticals.

[33] Keck T., Leiacker R., Riechelmann H., Rettinger G. (2000). Temperature Profile in the Nasal Cavity. Laryngoscope.

[34] ICH (2003). Stability Testing of New Drug Substances and Drug Products Q1A(R2).

[35] Aguilera-Garrido A., Molina-Bolívar J.A., Gálvez-Ruiz M.J., Galisteo-González F. (2019). Mucoadhesive Properties of Liquid Lipid Nanocapsules Enhanced by Hyaluronic Acid. J. Mol. Liq..

[36] Guarise C., Acquasaliente L., Pasut G., Pavan M., Soato M., Garofolin G., Beninatto R., Giacomel E., Sartori E., Galesso D. (2023). The Role of High Molecular Weight Hyaluronic Acid in Mucoadhesion on an Ocular Surface Model. J. Mech. Behav. Biomed. Mater..

[37] Laffleur F., Netsomboon K., Erman L., Partenhauser A. (2019). Evaluation of Modified Hyaluronic Acid in Terms of Rheology, Enzymatic Degradation and Mucoadhesion. Int. J. Biol. Macromol..

[38] Mattheolabakis G., Milane L., Singh A., Amiji M.M. (2015). Hyaluronic Acid Targeting of CD44 for Cancer Therapy: From Receptor Biology to Nanomedicine. J. Drug Target..

[39] Peach R., Hollenbaugh D., Stamenkovic I., Aruffo A. (1993). Identification of Hyaluronic Acid Binding Sites in the Extracellular Domain of CD44. J. Cell Biol..

[40] Yang Y.-T., Chen C.-T., Yang J.-C., Tsai T. (2010). Spray-Dried Microparticles Containing Polymeric Micelles Encapsulating Hematoporphyrin. AAPS J..

[41] Dattani S., Li X., Lampa C., Barriscale A., Damadzadeh B., Lechuga-Ballesteros D., Jasti B.R. (2025). Development of Spray-Dried Micelles, Liposomes, and Solid Lipid Nanoparticles for Enhanced Stability. Pharmaceutics.

[42] Fatnassi M., Jacquart S., Brouillet F., Rey C., Combes C., Girod Fullana S. (2014). Optimization of Spray-Dried Hyaluronic Acid Microspheres to Formulate Drug-Loaded Bone Substitute Materials. Powder Technol..

[43] Palumbo F.S., Agnello S., Fiorica C., Pitarresi G., Puleio R., Loria G.R., Giammona G. (2017). Spray Dried Hyaluronic Acid Microparticles for Adhesion Controlled Aggregation and Potential Stimulation of Stem Cells. Int. J. Pharm..

[44] Horvát S., Fehér A., Wolburg H., Sipos P., Veszelka S., Tóth A., Kis L., Kurunczi A., Balogh G., Kürti L. (2009). Sodium Hyaluronate as a Mucoadhesive Component in Nasal Formulation Enhances Delivery of Molecules to Brain Tissue. Eur. J. Pharm. Biopharm..

